# The driving factors behind urban communities’ carbon emissions in the selected urban villages of Jakarta, Indonesia

**DOI:** 10.1371/journal.pone.0288396

**Published:** 2023-11-10

**Authors:** Fatmah Fatmah

**Affiliations:** Disaster Management Study Program, School of Environmental Science Universitas Indonesia, Jakarta, DKI Jakarta Province, Indonesia; Universiti Teknologi Petronas: Universiti Teknologi PETRONAS, MALAYSIA

## Abstract

As there is a high number of urban communities and residential activities like electricity uses, transportation, food consumption, garbage production, and knowledge on climate change and carbon emissions, these affect the resulting carbon emissions in the city. However, the studies learn about the association between household and human activities, socio-demographic characteristic, carbon emissions and climate change knowledge on urban communities carbon emissions at Jakarta are still rare. This study aimed to assess whether daily human behavior, demographic variables, climate change knowledge, and carbon emissions knowledge affecting carbon emissions of urban communities. A cross-sectional study design was performed via a structured questionnaire survey and a *jejakkarbonku* application towards 200 respondents who were living in the two selected urban villages of Jakarta, Indonesia (Jatinegara and East Cengkareng). The results revealed that the mean urban communities’ carbon emissions in the region were 4.4 tonnes CO_2_ eq/th. Electricity consumption in the kitchen and dining room produced the most carbon emissions (38.6%), and waste disposal activity produced the least (0.68%). Urban carbon emissions affected by employment status and income level, but not according to food consumption, garbage production, climate change and carbon emission knowledge. Climate change and carbon emissions knowledge influenced by marital and employment status. Although not significant, in theory, these two types of knowledge contribute indirectly to carbon emissions. Further research on the low carbon diet, intervention studies to increase deep awareness on the carbon emissions and climate change is needed to develop strategies reducing carbon emissions at urban communities.

## Introduction

Decarbonized societies are crucial for reducing global carbon emissions that will, in turn, mitigate climate change. As more than half of the global population currently lives in urban areas, large cities and urban communities play a vital role in the transition to a decarbonized society. Mobility, housing, food consumption, consumer goods, and recreation behaviors all contribute to increase carbon emissions in large cities. A study conducted by the Institute for Essential Services Reform (IESR) in 2010, employed a carbon footprint calculator tool revealed that urban patterns, lifestyles, and energy consumption contribute to carbon emissions [1]. However, until recently, only few studies have explored the role of urban behavioral patterns or lifestyle on the total expenditure of carbon emissions mainly in the developing countries such as Japan, China, Indonesia, and Malaysia [2–6]. The limitation of five previous studies were did not analyze the relationship between climate change and carbon emissions knowledge and urban human communities’ carbon emissions. In addition, there were a limited number of carbon emissions and urban human lifestyle studies in Indonesia [7–11]. These studies focused on the factors contributing to the carbon emissions from income, electrical power, electricity consumption from household appliances, and not with the climate change and carbon emission knowledge. These two knowledge are required to develop educational program on climate change and to change human behavior to be low carbon.

Some preliminary studies mentioned that the most expenditure of urban lifestyle contributing to carbon emissions was electricity consumption and transportation in some developing countries such as India, China, and Egypt [4] and Japan [2]. Ahmad and Felix (2019) stated that the income and urbanization were the most contributors of house-hold carbon footprints in India [12]. Energy consumption level influenced by population density in transportation and electricity consumption in buildings, which in turn changes the carbon emissions level [13]. Various human activities have contributed to increasing greenhouse gas and carbon emissions in the earth’s atmosphere, resulting in global climate change, which affects the environment, the economy, and human health. The Intergovernmental Panel on Climate Change (IPCC) lists some of these activities, which includes the use of fossil fuels for cooking; the use of electrical devices; modes of transportation; bottled water consumption; animal-based food consumption as a source of protein; inorganic waste production; and the travel patterns of urban communities [14]. The impact of increased carbon emissions on human health includes mortality from extreme local weather changes, respiratory distress, and cardiovascular diseases (CVDs), along with increased risk of disease spread via airborne and animal vector transmission, particularly from rodents. Meanwhile, environmental impact takes the form of increased incidence of floods, forest fires, and infrastructure damage. A 2009 UK study demonstrated that individual lifestyles determine the quantity of carbon emissions produced because lifestyle affects individual consumption patterns [15].

Social factors, such as income and education, also play a role in determining carbon emissions expenditures [15]. Wynes & Nicholas (2018) reported that there were four human behaviors that can reduce carbon emissions: avoiding animal-based food consumption and shifting to plant-based (vegan) food consumption; reducing air transport; reducing motor vehicle usage; and having fewer children [16]. Individual behaviors that can reduce carbon emissions include reducing red meat consumption; shopping at traditional markets or local grocers; using bicycles for local transportation; shopping for fruit according to season to reduce waste due to damaged or rotten fruits; growing one’s own groceries; and reducing the consumption of carbohydrate-rich foods [17]. Research has demonstrated that beef and lamb are the foods that produce the greatest carbon emissions per kg [18]. On the other hand, a low-carbon diet can minimize emissions from production, packaging, processing, transportation, and food waste [19]. Factors influencing carbon emissions at urban communities are urbanization expansion, energy and electricity consumptions, income, industry sector, population size, family size, age structure, and population density.

Indonesia as one of developing countries in the world has a high on urbanization rate like Jakarta as nations’s capital city. It led to environmental pollution and high carbon emissions due to increasing private transportation and electricity consumption. Some studies stated that urbanization process in developing countries will gain the energy consumption and finally it will increase carbon emissions. Indonesia is home to several megacities, such as the Jakarta metropolitan area), total carbon emissions are increasing. Jakarta has ranking third in the top five cities in Indonesia in terms of the highest air quality index with four of its component cities [20]. During the COVID-19 pandemic, Jakarta succeeded in reducing Greenhouse Gas (GHG) emissions by 1.9–3 million tonnes. The current Jakarta government has declared it their mission to achieve a low carbon society by 2050 and to reduce carbon emissions by 30–50% by 2030 [21]. There are four main contributors to carbon emissions in Jakarta. The first is transportation with 46%, power generation sector 31%, the manufacturing industry 8%, and residential or household waste emissions 6%. The industrial sector has three sources of GHG, namely energy use (40%), and the remainder came from process technology and industrial waste which lead to CO2 [22]. Therefore, it is necessary to further assess the determinants of carbon emissions in urban communities. Furthermore, there is still little research and literature focusing on the impacts of urbanization and lifestyle on carbon emissions in Indonesia [7–11]. The current study has contribution to add to the treasury of studies on factors affecting carbon emissions in Jakarta’s urban communities in Indonesia. The aim of the present study was to explore the association between certain demographic characteristics, daily activities, climate change knowledge, and carbon emissions knowledge and total carbon emissions at two selected areas of Jakarta’s urban communities. The current study can provide important baseline data to develop the intervention program reducing carbon emissions from climate change and carbon emissions perspectives at urban communities.

## Materials and methods

### Study design

This cross-sectional study was performed on 200 respondents of a survey interview who were selected using simple random sampling. Ethical clearance for this study was obtained from the Health Research Ethics Commission of the Universitas Pembangunan Nasional “Veteran” Jakarta (No. 353/VI/2022/KEPK). All respondents in this study had signed a written inform consent form before participating in face-to-face interviews in the second week of July 2022.

### Population and sample

The inclusion criteria for the respondents were men and women aged between 18 to 75 years who were either working or unemployed, who had graduated from at least senior high school, and had been living for a minimum of one year in one of the two selected urban villages, Jatinegara Urban Village, Cakung Subdistrict, East Jakarta and East Cengkareng Urban Village, Cengkareng Subdistrict, West Jakarta. Both urban villages are located in DKI Jakarta. Jatinegara Urban Village was selected as one of the study locations by Purposive Sampling due to its poor air quality who has the highest level of air pollution in Jakarta according to the Urban Air Quality Evaluation [23]. The minimum sample size was calculated using the proportion estimation with absolute precision hypothesis test. This study used a two-sided significance level of 0.05. Thus, a sample size of n = 200 was needed to reach 95% statistical power [24].

### Variables measurement and research framework

Household play role as drivers in the total carbon emission such as increasing income, household composition and size, urban location, diet and type of energy supply [25]. There were three domains driving total carbon emission, namely housing, transportation, and food [9,13]. Some studies indicated that working from home, recreation, sport, and leisure pursuits can reduce average carbon emissions [26–28]. Studies indicated that activities such as spending time with friends and family in and around the home will enhance human wellbeing and having low carbon emissions [29,30].

**Fig 1:**
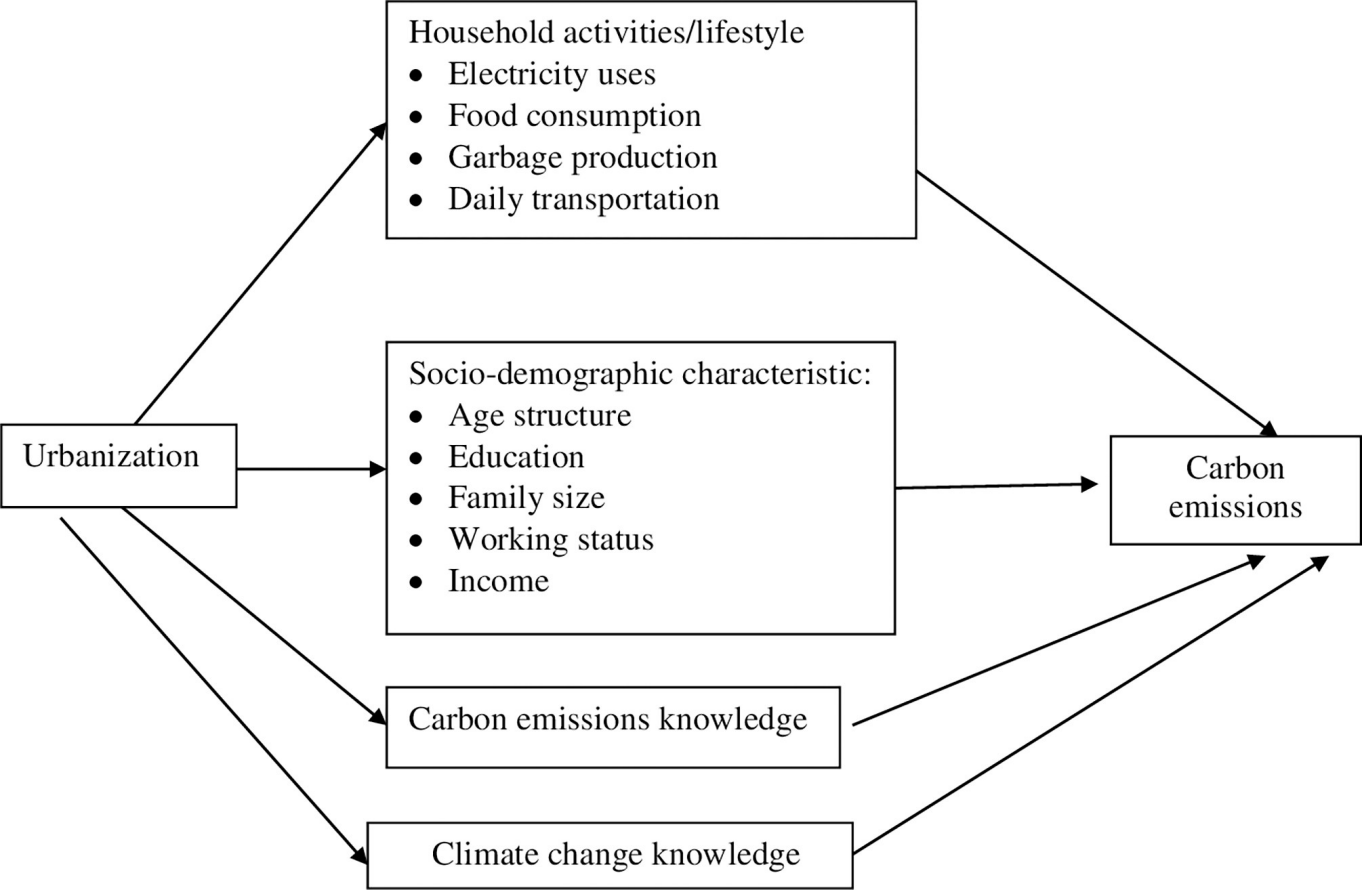
Research framework.

The study focused on the household and human activities (electricity uses, food consumption, garbage production, daily transportation), socio-demographic characteristic (age structure, education, family size, working status, and income), carbon emissions knowledge, and climate change knowledge were the independent variable contributing to the total carbon emissions as the dependent variable (Fig 1). As indirect factor, urbanization also has an indirect impact on carbon emissions through household activities and lifestyle [13].

### Data collection

Primary data collection was performed through home visits in July 2022 by five trained enumerators who were experienced in conducting survey interviews using a structured questionnaire. The items in the questionnaire were categorized into socio-demographic characteristics (marital status, age, education, employment, monthly income, and ownership of electronics); climate change knowledge (definition, causal factors, current and felt impacts of climate change, most vulnerable regions to climate change, the efforts of the Jakarta government to reduce the impact of climate change, including prevention efforts); and knowledge of carbon emissions (definitions, contributing factors, key sectors contributing to carbon emissions, and similarities between carbon emissions and climate change). Jejakkarbonku.id, an application developed by the IESR in 2011, was used in the current study as a tool to calculate the strength of each greenhouse gas produced in each human activity, including means of transportation; use of electronic items/devices and lamps (in the bedroom, living room, bathroom, kitchen, and dining room); and frequency of food consumption over the previous seven days. The unit used in this carbon footprint calculator is the equivalent to grams of CO_2_ per day for individual analysis [1].

### Statistical analysis

Data were analyzed using univariate and bivariate analyses. The frequency distributions, mean, and standard deviation of independent variables, i.e. socio-demographic characteristics, climate change knowledge, carbon emissions knowledge, and carbon emissions as the dependent variable, were the outputs of the univariate analysis. A dependent t-test was used to determine if there was a change from one measurement to another within-group measurement. A p-value of less than 0.05 was considered statistically significant. The climate change and carbon emissions knowledge were processed in the following way. Each correct answer on the items measuring knowledge of climate change and knowledge of total carbon emissions were scored 1. Incorrect answers or *do not know* responses were scored 0. Then, both the knowledge level groups were classified into two categories: namely, low level (less than the mean score) and sufficient level (more than or equal to the mean score). All scores were added, and the total correct answer score was calculated as a percentage. The mean and standard deviation of both types of knowledge (climate change and carbon emissions) were calculated to perform bivariate analysis with socio-demographic characteristics and total carbon emissions using an independent t-test.

## Results

### Demographic characteristics, transportation, food consumption, and waste disposal

The majority of respondents were in the young adult age group, ranging from 25 to 43 years old. The proportion of the younger group (under 25 years old) was almost the same as that of the middle-aged group (44–59 years). Only a small number of older people participated in the study. More than three-quarters of the respondents had completed senior high school, and the percentage of respondents who completed higher education (bachelor/masters/doctorate) was small. Half of the total respondents were unemployed and of those with jobs, most worked as traders, were self-employed, and/or were private employees. The majority of respondents had a monthly income of less than $300. However, some did not know the amount of money that their family earned. More than half of all respondents were married, and only a small percentage were widowed. It was found that working status is linked to the total expenditure of carbon emissions (Table 1).

**Table 1:** Socio-demographic characteristic of respondents.

Variable		Number (person)	Proportion (%)
Age (years old)			
	Youth (13–24)	38	19.0
	Young (25–43)	79	39.5
	Middle age (44–59)	67	33.5
	Older people (60–74)	16	8.0
	Mean + SD	40.6 ± 13.8	
Last education			
	Did not finish senior high school	22	11.0
	Finished senior high school	154	77.0
	Academy	10	5.0
	Bachelor degree	12	6.0
	Master/Doctoral degree	2	1.0
Main job status:			
	No job	102	51.0
	Having job		
	Private sector employee	22	11.0
	Trader	37	18.5
	Entrepreneur	30	15.0
	Others	9	4.5
Monthly household income			
	Less than US 300	134	67.0
	More than or equal to US 300	42	21.0
	Do not know	24	21.0
	Mean + SD	327.2 ± 217.6	

The mean total carbon emissions from the means of transportation and consumption of carbohydrate, protein, fat, vitamin, and mineral-rich foods were similar. However, this was not the case with the individual total carbon emissions from garbage disposal activities. Gasoline-fueled motors were the most frequently used vehicles by respondents, followed by gasoline-fueled- car and solar-fueled-bus. Foods that were widely consumed by respondents were rice, eggs, poultry, meat, fish, tofu, and tempeh, while beef, lamb, and seafood had been rarely consumed by respondents over the previous week. The quantity of plastic waste disposed was twice as much as paper waste (Table 2).

**Table 2:** Frequency distribution of transportation use and distance/week, food consumption per week, and waste disposal (garbage)/week.

Variable		n	%	Mean ± SD		
				Distance/week (km)	Frequency/week (time)	Total carbon missions (tonCO2-eq/year/capita)
Total carbon emissions of three activities						4.43 ± 4.30
Transportation						0.85 ± 3.61
	Gasoline motorcycle	178	89	38.0 ± 48.3		
	Gasoline car	24	12	69.9 ± 75.9		
	Solar bus	10	5	82.9 ± 59.1		
Food consumption						0.82 ± 0.78
	Rice	198	99.0		6.9 ± 0.3	
	Bread	94	47.0		2.3 ± 1.6	
	Noodle	134	67.0		2.2 ± 1.3	
	Egg	195	97.5		5.4 ± 2.1	
	Seafood	34	17.0		1.4 ± 0.5	
	Fish	172	86.0		2.9 ± 1.7	
	Poultry	182	91.0		2.8 ± 1.8	
	Beef	71	35.5		1.5 ± 1.0	
	Sheep	5	2.5		0.8 ± 0.4	
	Tofu	191	95.5		4.3 ± 1.8	
	Tempeh	192	96.0		4.6 ± 1.8	
	Vegetable	29	14.5		5.7 ± 1.9	
	Fruits	4	2.0		4.5 ± 1.9	
	Coffee	95	47.5		4.7 ± 2.0	
	Tea	115	57.5		4.7 ± 2.0	
	Milk	93	46.5		3.4 ± 2.3	
Waste disposal garbage						0.03 ± 0.07
	Plastic waste				0.6 ± 0.7	
	Paper waste				0.3 ± 0.5	

### Household electronic use in the rooms and total carbon emissions

Household activities that produce the most carbon emissions in this study comprise the use of various electronic appliances and equipment, such as fluorescent lamps, incandescent lamps, Light-Emitting Diode (LED) lamps, refrigerators, gas stoves, inverter refrigerators, non-inverter refrigerators, dispensers, air fryers, rice cookers, ovens, microwaves, and mixers. Activities such as electrical energy consumption (use of lamps, kitchen utensils, shavers, and electronic devices), daily waste production (organic waste, paper, bottled drinking water bottles), and use of transportation (motor vehicles and cars) can produce CO_2_ (Table 3). LED lights used a combined total of 300 watts, and each home used an average of 7.5 LED bulbs on a given day. They were most widely used for 7.5 hours a day in all spaces within the respondents’ homes. Inverter air conditioners with power ratings of 300 watts were most often used in the living room but not in the sleeping areas. The majority of respondents used the television in the living room, rather than the bedroom. Non-inverter refrigerators, gas stoves, and rice cookers were widely used by respondents in the kitchen and dining room. The highest total carbon emissions were found in the kitchen and dining room, while the lowest total carbon emissions were found in the bathroom (Table 3).

**Table 3:** Frequency distribution of use electronic goods use based on the type of room in the house.

Type of electronic goods	Living room					Bed room					Kitchen and dining room					Bathroom				
	n	%	amount (pieces)	power (watt)	duration (hours)	n	%	amount (pieces)	power (watt)	duration (hours)	n	%	amount (pieces)	power (watt)	duration (hours)	n	%	amount (pieces)	power (watt)	duration (hours)
Fluorescent lamps	55	27.5	1	15	10	52	26.0	2	15	10	37	18.5	1	15	10	35	17.5	1	10	24
Incandescent lamps	16	8.0	1	12.5	7.5	15	7.5	2	10	8	16	8.0	1	10	7.5	28	14.0	1	5	24
LED lamps	135	67.5	1	16	7.5	134	67.0	1.5	16	7.5	129	64.5	1	16	7.5	133	66.5	1	16	12
Inverter air condition	14	7.0	1	300	8	11	5.5	1	300	5	0	0	0	0	0					
Non inverter air condition	8	4,0	1	350	8	28	14.0	1	350	8	0	0	0	0	0					
Fan	156	78.0	1	53	8	135	67.5	1	53	7	0	0	0	0	0					
TV	171	85.5	1	72	4	23	11.5	1	72	3	0	0	0	0	0					
Inverter refrigerator											26	13.0	1	60	24					
Non inverter refrigerator											162	81.0	1	90	24					
Gas stove											190	95.0	1	0	2					
Electrical stove											3	1.5	1	310	1.75					
Rice cooker											184	92.0	1	310	3					
Dispensers											78	39.0	1	245	24					
Oven											7	3.5	1	295	1					
Microwave											10	5.0	1	628	0.5					
Mixers											9	4.5	1	185	0.5					
Blender											63	31.5	1	215	1					
Air fryer											2	1.0	1	999	2.5					
Total carbon missions (tonCO2-eq/year/capita)		0.35 ± 0.30					0.43 ± 0.60					1.7 ± 1.4					0.08 ± 0.09			
							

### Climate change and carbon emissions knowledge with total carbon emissions

Climate change knowledge was significantly different in regard to different age groups, employment status, marital status, and monthly income levels (Table 4). There were also significant differences in carbon emissions knowledge based on gender, highest education levels, marital status, and employment. The total carbon emissions produced by individuals differed significantly in respondents with different employment status and income levels. Total carbon emissions were significantly associated with climate change knowledge but not to carbon emissions knowledge. Mean total carbon emissions from the respondents with a sufficient level of knowledge were slightly lower than that of the respondents with a low level of knowledge. Total individual carbon emissions in the study exhibited a statistically significant relationship with climate change knowledge, working status, and income per month. This study demonstrates that a low level of knowledge of climate change results in more total emissions than a sufficient level of knowledge. The majority of respondents had a low level of climate change knowledge, despite most respondents possessing a sufficient level of carbon emissions knowledge. Low levels of climate change and carbon emissions knowledge resulted in lower total carbon emissions than when both types of knowledge were higher. There was a significant difference in mean total individual carbon emissions when compared among different levels of knowledge of climate change (p = 0.003). In contrast, there was no relationship between the knowledge level of carbon emissions and mean total individual carbon emissions (p = 0.19).

**Table 4:** Difference of climate change knowledge, carbon emissions knowledge, and total carbon emissions based on the socio-demographic characteristic.

Variable		n	Knowledge on Climate Change (range 0–100)		Knowledge on Carbon Emissions (range 0–100)		Total Carbon Emissions (tonCO2-q/year/capita)	
			Mean ± SD	p	Mean ± SD	p	Mean ± SD	p
Sex								
	Man	61	32.4 ± 18.0	0.235	44.9 ± 24.5	0.002**	4.9 ± 5.9	0.279
	Woman	139	31.1 ± 16.2		33.5 ± 22.4		4.2 ± 3.3	
Age group (years old)								
	Youth (13–24)	38	37.9 ± 21.5	0.007**	42.4 ± 23.7	0.336	3.9 ± 1.8	0.483
	Young (25–43)	79	32.9 ± 16.9		36.1 ± 24.2		4.9 ± 4.1	
	Middle age (44–59)	67	30.4 ± 13.6		36.6 ± 22.2		4.4 ± 5.7	
	Older people (60–74)	16	21.4 ± 10.3		30.5 ± 24.9		3.5 ± 2.2	
Final education								
	Middle (high school maximum)	176	31.8 ± 17.6	0.446	36.4 ± 23.9	0.024*	4.4. ± 4.5	0.898
	High (academy/master degrees)	24	34.6 ± 17.6		41.2 ± 21.0		4.5 ± 2.2	
Marital status								
	Married	145	30.3 ± 15.7	0.006**	34.8 ± 23.3	0.015*	4.8 ± 4.9	0.199
	Single	36	40.2 ± 20.5		47.2 ± 22.2		3.8 ± 1.7	
	Widow/widower	19	30.5 ± 13.3		34.2 ± 24.2		3.2 ± 1.4	
Main job status								
	No job	111	36.5 ± 18.4	0.005**	40.7 ± 24.1	0.007**	3.7 ± 1.6	0.002**
	Private employee	22	31.3 ± 16.2		38.6 ± 22.1		4.9 ± 2.3	
	Trader	37	30.9 ± 16.2		32.4 ± 20.3		3.6 ± 2.2	
	Entrepreneur	30	33.6 ± 17.0		45.4 ± 24.0		7.3 ± 9.6	
Monthly household income								
	< US 300	134	29.4 ± 14.6	0.004**	35.5 ± 22.9	0.227	3.7 ± 2.6	0,001**
	> US 300	42	36.4 ± 17.7		42.6 ± 24.6		6.8 ± 7.6	
	Do not know	24	39.6 ± 22.9		35.9 ± 24.5		4.2 ± 2.3	

* p<0.05

** p<0.01.

Variable of sex and final education with **independent t test**: Other variables with **One-Way ANOVA test**.

## Discussion

The mean total individual carbon emissions in this study are slightly above the national mean total carbon emissions for Indonesia in 2019 of 1.1 tonnes CO_2_-equivalent per capita. The total carbon emissions in Indonesia is relatively low compared to the emissions in China and Japan [20]. This lower value is consistent with the government of Indonesia’s commitment to reduce Green House Gas (GHG) emissions to 1240 million tonnes of CO_2_ equivalent by 2030 and to 540 million tonnes CO_2_ equivalent by 2050. It is expected that Indonesia will achieve zero emissions by 2060 or sooner [31]. Climate change phenomenon is closely associated with GHG because GHG levels affect the occurrence of global warming, climate change, and hydrometeorological disasters [32], while climate change itself is affected by individual behaviors. Mikhaylov and Moiseeve (2020) demonstrated that a decrease in transportation activities and household activities reduced the production of GHG [33].

Urbanization process has negative impact on the carbon emissions because population density increase direct and indirect energy consumption at household. Urbanization at developing countries has more energy consumption through household activities, industrial activities, and transportation which will increase carbon emissions for developing countries [34,35]. This is different with urbanization in develop countries who reduce carbon emissions due to increasing environmentally friendly awareness by government with their policy and programs to mitigate carbon emissions and global warming ([13].

Some studies explored the association human lifestyle at urban city and carbon emissions in developing countries such as Japan, China, Indonesia, and Malaysia [2–6]. Change in lifestyle covering housing, food, and mobility reduced carbon footprints in Japan communities [2]. Transportation-related emissions and cooking fuels emissions were the biggest contributor of carbon emissions in Indonesia [3]. Electricity consumption and transport are the largest contributors to the total carbon emissions in developing countries [4]. Urbanization in developing countries through economic scale, energy consumption and transportation increase carbon emissions [5]. Urban households of Malaysia contributed more carbon emissions compared to rural households [6]. These five studies highlight the contribution of household and indirect consumptions to carbon emissions which differed with the present studies. The main thing distinguished these studies with the current study was the role of climate change knowledge on the urban carbon emissions. The limitation of these studies are that other factors such as climate change and carbon emissions knowledge were also significant points of concern in carbon emissions research. Besides that, the number of these two research topic related to urban carbon emissions in Jakarta is limited.

Urban communities’ carbon emissions in the study originated from four major domains: electricity consumption in the kitchen and dining room; private transportation from gasoline-fueled motorcycles; food consumption; and waste disposal. Motor vehicle emissions, electricity use, industrial waste, deforestation, and food waste trigger global warming and climate change. These human activities increase the concentration of CO2, which is the most significant contributor to climate change and global warming [36]. Transportation and industrial are the primary sources in urban areas because they produce sources of air pollution, namely CO2, CH4, N2O, CO, HC, and particulate matter (PM). Road transportation (motor vehicle, car, bus, trucks) produces 91% of total carbon emissions in urban cities [37]. Jakarta is already trying to reduce these by switching private gasoline-fueled vehicles to electricity; increasing public transportation; making public transportation facilities even more comfortable, such as the LRT (Light Rapid Transportation) and the MRT (Mass Rapid Transportation); improving emission standards for new vehicles; and evaluating local policies on reducing fuel consumption and emission levels [38].

Jakarta has introduced green building standards, green architecture, and residential areas and neighborhoods design and planning to reduce carbon emissions and promote sustainable living. An integrated approach to different demands (social, architectural, artistic, economic, sanitary, and hygienic) must be considered. A comprehensive approach to reduce carbon emissions must include material production, logistics, design, and construction processes. Green architecture focuses on employing sustainable construction methods; reusing environmentally safe building materials; reducing energy expenditure during construction; and designing buildings to be more energy efficient in the long run. A transition to sustainable architecture approaches in urban planning is necessary to meet urgent climate goals, [39] as building codes and energy efficiency standards impact carbon emissions in urban communities. Building energy codes provide a tool for governments to mandate the construction and maintenance of low-energy buildings, reduce energy use, and cut carbon emissions [40].

In the current study, climate change knowledge, working status, and income per month has effects on the total carbon emissions. A low level of climate change knowledge resulted in more total emissions than a sufficient level of knowledge. In addition, total carbon emissions are not directly related to climate change knowledge. Knowledge is one of several factors affecting the behavior change that leads to awareness and then to action [41]. The level of climate change knowledge affects people’s lifestyles and behavior and thus, affects total carbon emissions. Knowledge of climate change plays a role in the expenditure of individual carbon emissions. This result is consistent with other studies stated that climate change knowledge facilitated low-carbon behavior [42] and associated well with bigger awareness and positive attitudes towards the climate [43].

Working status is linked to the total expenditure of carbon emissions in the current study. More time spent indoors by non-working individuals contributes to an increase in total carbon emissions [44]. The majority of surveyed community carbon emissions come from the middle-income groups, as their lifestyles significantly impact the overall carbon emissions. Most respondents had total monthly income less than US 300 which is equal to Provincial Regular Minimum Wage for Jakarta [45]. Middle to high family income can increase carbon emissions expenditures due to an increased use of electronic equipment, electric transportation, and so on. [46]. Furthermore, a human’s lifestyle significantly contributes to global warming because it determines the number of goods used, the amount of paper and electricity used, and various means of transportation used. Therefore, low-carbon lifestyle patterns are needed, such as reducing electronic goods use and increasing the community capacity for implementing energy efficiency importance in home and work environments.

Although in the current study, age group does not correlate with significant differences in total carbon emissions, the young adult and middle-aged group’s (25–59 years) carbon emissions were greater than those of the younger age group (13–24 years) and the elderly group (65–74 years). This finding is consistent with a study in the Indonesian working age population that demonstrated that productive age groups (15–59 years) produced higher total carbon emissions than the non-productive age groups (0–15 years and over 65 years) [3]. The current study’s finding seems to contradict with other study in the urban household at Yangtze River Delta, China, which showed that carbon emissions will increase slightly as people get older [47]. The study found that older people travel less, have less private car use, and reduce their food consumption but increase the time spent at home. As spending more time at home increases electricity and heat consumption, this results in higher carbon emissions. These differences caused by the characteristic of city, number of sample, and geographical location.

Climate change knowledge in the present study is inversely related to age. The lower the age, the higher the knowledge score. This is in contrast with a study conducted in Yogyakarta’s adolescent respondents, which demonstrated this low knowledge of climate change [48]. The second study undertaken in Yogyakarta with 508 teenagers at senior high school. They knew that the climate change caused by a natural process. The current study also found that most respondents learn about climate change from visual media. Human behaviors are the main cause of environmental changes due to climate change, such as hotter weather and more rain. However, low levels of climate change knowledge were still dominant among respondents in this present study. This finding was corroborated by the low level of knowledge found among coastal communities of North Minahasa District [49]. However, the finding of present study seems to be contrary to other study [50]. The result stated that climate change knowledge level did not influence individual carbon footprint. The results difference from the two studies was due to method and sample differences. It seems that climate change is a phenomenon that is difficult for the general public to understand, though individual perceptions and climate change knowledge can have an effect on mitigating and adapting behaviors to reduce the climate change impacts risks. Climate change concerns human lives and affects people’s lifestyles related to house building, transportation, energy use, consumption, and food waste, thereby affecting total carbon emissions [51].

One key finding of the current study is that carbon emissions knowledge differed significantly among participants with different genders, education levels, employment status, and marital status. This is consistent with Peek et al.’s (2018) study, which found that higher education levels are linked to cognitive development, problem-solving abilities, knowledge, and perceptions of the risk of environmental damage [52]. Gender and employment status have also been linked to an understanding of carbon footprints in Spanish population [40]. Previous studies have demonstrated that men have higher environmental knowledge than women [53]. Furthermore, marital status significantly influences climate change information, including climate change awareness and carbon emissions knowledge, especially among women [54,55]. This may be relevant when developing interventions to increase carbon emissions knowledge. Also, as carbon emissions knowledge has been found to have a close relationship with an individual’s carbon emissions status, increasing knowledge will improve an individual’s carbon emissions status [56].

In this present study, total carbon emissions were significantly associated with climate change knowledge, but not to GHG knowledge. Mean total carbon emissions of the respondents with sufficient level of knowledge were found to be slightly lower than that of the respondents with a low level of knowledge. In addition, climate change knowledge affected by age groups, family income levels, and employment status. This is consistent with studies in Bangladesh that demonstrated the relationships between climate change knowledge and age, income, and employment status [57,58].

Several efforts need to be made by local governments to reduce total carbon emissions of urban communities. **First**, governments should implement walkable cities and sustainable and climate-resilient cities through transportation efficiency with compact development and public space design (with provision of pedestrian paths and city parks as needed, thereby reducing waste) [58]. **Second**, forms of government–private partnerships should reduce urban carbon emissions through renovations of sewage management systems; financial/budgetary assistance; technology cooperation agreements; capacity building of human resources through training and seminars/webinars; and research and community service, recording and reporting, and so on [59]. **Third**, local government policies and regulations should aim to reduce urban carbon emissions through the transportation sector (which is the third largest contributor to city carbon emissions in Jakarta at around 33%). The policies stipulated include setting low ticket prices for the public from government subsidies for buses and trains; subsidizing the cost of buying electric motorized vehicles; promoting environmentally friendly (electric) public transportation; and standardizing minimum carbon emission limits for motorized vehicles [60]. Mitigation and adaptation are the most effective approaches in developing urban carbon emission reduction policies because these two approaches are related to the resilience of low-carbon cities [61]. The potential benefits of environmentally friendly transportation are to develop healthier cities that lead to more carbon-neutral environments. The drawback is slow public acceptance because they need to get used to it, and the price is high. The most effective communication strategies for engaging residents, businesses, and local governments in efforts to reduce carbon emissions in urban communities are to employ authoritative scientific information, convey the problems and solutions, and mobilize action [62]. **Fourth**, state budget allocation for the green economy (to improve environmental quality, increase disaster resilience, and reduce climate change) and low-carbon development drive economic growth and improve the social welfare of urban communities [63].

## Conclusions

Electricity consumption in the kitchen and dining room and private transportation from gasoline-fueled motorcycles and cars have been major contributors to the urban communities’ carbon emissions in the selected areas of Jakarta, Indonesia. A set of demographic characteristics (gender, education, marital status, and employment status); climate change knowledge; and the purchasing of seasonal fruit in the local traditional market are driving factors of urban communities’ carbon emissions in the field. Climate change knowledge, working status, monthly income, and the habit of shopping from local markets all influence urban communities’ carbon emissions significantly. A differential interdependence was found between climate change knowledge and total individual carbon emissions but not carbon emissions knowledge. Climate change knowledge significantly differs by age, employment status, marital status, and income, while carbon emissions knowledge significantly differs by gender, highest education, marital status, and working status. An innovation of the current study compared to the existing research is the *jejakkarbonku* calculator application developed by IESR to analyze factors affecting total carbon emissions in some selected areas of urban Jakarta. Along with the benefit of increasing knowledge and awareness of this calculator’s users, it is expected that users will reduce their carbon emissions by reducing or eliminating their insignificant daily activities. In addition, the present study also calculates the total carbon emissions based on the consumption of foods rich in carbohydrates, proteins, and fats, as well as on household electronic use in each room at home.

This study is limited in that **first**, the measurement of daily food consumption in the week prior to respondents being interviewed only included the frequency of consumption and portion size (weight) but did not include analyses of carbohydrates, proteins (vegetable and animal), and fatty rich foods nutrient consumption. However, the accuracy of obtaining data on the duration of appliance and automobile use and related energy consumption is quite high. This can be seen from the calculator calculation and manual calculation being the same. The researcher had validated the results of individual food records (except for the food portion size) from the *jejakkarbonku* calculator with the Food Frequency Questionnaire (FFQ).

We recommend categorizing the types of low-carbon diet foods consumed (fruits, vegetables, etc.) in the jejakkarbonku.id tracing application to calculate the proportion of individuals who have employed a food consumption pattern that reduces individual carbon emissions, which is carbohydrate- animal protein-rich foods. **Second**, jejakkarbonku.id also does not include a count value of the volume of plastic and paper waste disposed, such as the weight of plastic waste in one bag in kg, the weight of 50 pieces of paper waste, and so on. **Third**, the distance travelled by respondents when using diesel buses, electric buses, airplanes, and electric trains is difficult to determine because there are no average estimates of the total distance in km provided. A specific exploratory study is crucial to assess the effect of low-carbon food dietary practices on the total individual emissions expenditure compared to high-carbon food diets. Studies on the relationship between the application of low-carbon diet activities in urban communities and the total expenditure of carbon emissions are also necessary to encourage the improvement of carbon emissions studies with healthy lifestyles in urban communities. **Fourth**, the selection of two study areas by purposive sampling who have the highest level of air pollution in Jakarta [11] can not generalize and represent the study results at Jakarta and Indonesia levels. However, characteristic of Indonesian communities carbon emissions in the current study was almost similar with Indonesian National Economic Survey (*SUSENAS*) [3], i.e. transportation sector affected the community carbon emissions.

## Supporting information

S1 File (PDF)

## References

[1] Institute for Essential Services Reform (IESR). Report of Indonesia’s carbon footprint calculator potential emission reduction through individual lifestyle changes. Jakarta: IESR; 2011.

[2] Koide RY, Lettenmeier M, Akenji L, Toivio V, Amellina A, Khodke A, et al. Lifestyle carbon footprints and changes in lifestyles to limit global warming to 1.5°C, and ways forward for related research. Sustainability Science 2021; 16: 2087–2099.

[3] Wulandari MD. Working age population and CO emissions in Indonesia: household approach. Proceeding on The International Conference on Data Science and Official Statistics (ICDSOS) 2022: 482–491.

[4] Connolly M, Shan Y, Bruckner B, Li R, Hubacek K. Urban and rural carbon footprints in developing countries. Environmental Research Letters 2022;17 (8): 1–10.35662858

[5] Chen F, Liu A, Lu X, Zhe R, Tong J, Akram R. Evaluation of the effects of urbanization on carbon emissions: the transformative role of government effectiveness. Frontiers in Energy Research 2022; 10 (): 2–12.

[6] Chik NA, Radam A, Rahim KA, Shamsudin MN. CO2 emission induced by households lifestyle in Malaysia. International Journal of Business and Society 2013;14 (3): 344–357.

[7] Hardiyanti V. Jejak karbon (carbon footprints) dari civitas akademika (Univesritas Atmajaya Yogyakarta). Skripsi. Yogyakarta: Universitas Atmajaya Yogyakarta; 2013.

[8] Indriyani L, Gandri L, Padang JL, Bana S. Carbon footprint analysis of household activities and CO2 reduction efforts in Pondambea Village, Kadia District, Kendari City. Journal of Soilscape and Agriculture 2020; 1(1): 15–21.

[9] Cahyono WE, Parikesit, Joy B, Setyawati W, Mahdi R Projection of CO2 emissions in Indonesia. Materials Today Proceedings 2022; S438–S444.

[10] Astari RG. Studi jejak karbon dari aktivitas pemukiman di Kecamatan Pademangan Kotamadya Jakarta Utara. Depok: FT UI; 2012.

[11] Suryati I, Hijriani A, Indrawan I. Estimation of greenhouse gas emission from household activities during the COVID-19 pandemic in Binjai City, North Sumatera. IOP Conf. Series: Earth and Environmental Science 2021; 896: 1–9.

[12] Ahmad S, Creutzig F. Spatially contextualized analysis of energy use for commuting in India. Environmental Research Letters 2019; 14: 1–11.35340667

[13] Chen F, Liu A, Lu X, Zhe R, Tong J., Akram R. Evaluation of the effects of urbanization on carbon emissions: the transformative role of government effectiveness. Frontier in Energy Research 2022; 10: 1–12.

[14] IPCC. Climate change 2022: impacts, adaptation and vulnerability working group II contribution to the sixth assessment report of the intergovernmental panel on climate change. UK: Cambridge University Press; 2022.

[15] Baiocchi G, Minx J, Hubacek K. The impact of social factors and consumer behavior on carbon dioxide emissions in the United Kingdom. Journal of Industrial Ecology 2010.

[16] Wynes S, Nicholas KA. The climate mitigation gap: education and government recommendations miss the most effective individual actions. Environmental Research Letters 2017; 12: 1–9.36204013

[17] Yang X., Zhihang Z, Huangyixin C, et al. Assessing the carbon emissions driven by the consumption of carbohydrate-rich foods: the case of China. Sustainability 2019; 11:1–15.

[18] Rippin HL, Cade JE, Ford LB, Benton TG, Hancock N, Greenwood DC. Variations in greenhouse gas emissions of individual diets: associations between the greenhouse gas emissions and nutrient intake in the United Kingdom. Plos One 2021;16(11):1–17. doi: 10.1371/journal.pone.0259418 34813623 PMC8610494

[19] Hjorth T, Ena H, Elinor H, et al. Changes in dietary carbon footprint over ten years relative to individual characteristics and food intake in the Västerbotten Intervention Programme. Scientific Reports 2020; 10 (20): 1–14. 31913331 10.1038/s41598-019-56924-8 PMC6949226

[20] IQ*Air*. World air quality report 2020. Swiss: IQair; 2020.

[21] Environmental Services of DKI Jakarta Province. Report on the Reduction of Greenhouse Gas Emissions in DKI Jakarta Province. Jakarta: Environmental Services of DKI Jakarta Province; 2019.

[22] Gatot S, Lailan S, Nining P. Estimation of emissions of equivalent carbon dioxide (Co2-Eq) equivalent due to changes in land use in Bogor Regency. Thesis. Bogor: Bogor Agriculture University; 2019.

[23] DKI Jakarta Provincial Government. Final Report of air quality monitoring of DKI Jakarta Province Year 2021. Bogor: IPB University; 2021.

[24] Ariawan I. Sample size and methods in health research. Depok: Faculty of Public Health Universitas Indonesia;1998.

[25] Druckman A, Jackson T. Understanding household as drivers of carbon emissions in book Taking Stock of Industrial Ecology. UK: Springer; 2016.

[26] Garra TO, Fouquet R. Willingness to reduce travel consumption to support a low-carbon transition beyond COVID-19. Ecological Economics 2022; 193: 1–17. 10.1016/j.ecolecon.2021.107297 PMC8608619 34840426

[27] Cerqueira EDV, Baumvol BM, Chevallier LB, Bonin O. Does working from home reduce CO2 emissions? An analysis of travel patterns as dictated by workplaces. Transportation Research Part D 2020; 83: 1–12.

[28] Druckman A, Gatersleben B. A time-use approach: high subjective wellbeing, low carbon leisure. Journal of Public Mental Health 2019; 18(2): 85–93.

[29] Druckman A, Buck I, Hayward B, Jackson T. Time, gender and carbon: A study of the carbon implications of British adults’ use of time. Ecological Economics 2012; 84: 153–163.

[30] Li Q, Chen H. The relationship between human well-being and carbon emissions. Sustainability 2021;13(5):1–12.

[31] Ministry of Environment and Forestry. Operational plan Indonesia’s FOLU Net Sink 2030. Jakarta: Ministry of Environment and Forestry; 2022.

[32] Patrianti T, Amin S, Retnowati WDT. Government risk communication on greenhouse gas emission reduction to tackle climate change. Jurnal Penelitian Komunikasi 2020; 24(2):156–170.

[33] Mikhaylov A, Nikita M. Global climate change and greenhouse effect. Entrepreneurship and Sustainability Issues 2020; 7(4): 2897–2913.

[34] Glaeser EL, Kahn ME. Chapter 56 Sprawl and Urban Growth in Handbook of Regional and Urban Economics (Elsevier) 2004; 2481–2527.

[35] Sadorsky P. The effect of urbanization on CO2 emissions in emerging economies. Energ. Econ. 2014; 41:147–153.

[36] European Commission. Causes of climate change. https://climate.ec.europa.eu/climate-change/causes-climate-change_en.{Accessed 30 December 2022).

[37] Azhaginiyal A, Umadevi G. System dynamics simulation modelling of transport, energy and emissions interaction. Civil Engineering and Architecture 2014; 2(4):149–165.

[38] Sukarno I, Matsumoto H, Susanti L. Transportation energy consumption and emissions-a view from city of Indonesia. Future Cities and Environment 2016; 2 (6): 2–11.

[39] Leyzerova A, Sharovarova E, Alekhin V. Sustainable strategies of urban planning. Proceedia Engineering 2016; 150: 2055–2061.

[40] Valence Gd. 2022. Building standards, energy codes and decarbonization. https://www.constructioneconomicsresearch.com/post/building-standards-energy-codes-and-decarbonisation. {Accessed 15 November 2022).

[41] Bray BJ, Cridge AG. Can education programmes effect long term behavioural change. Int J Innov Interdiscip Res 2013; 2: 27–3.

[42] Zhang J, Zepeng T, Zeyu J, et al. Effects of climate change knowledge on adolescents’ attitudes and willingness to participate in carbon neutrality education. Int J Environ Res Public Health 2022; 19: 1–16. doi: 10.3390/ijerph191710655 36078370 PMC9518399

[43] Van VAM, Steg L. Meta-analyses of factors motivating climate change adaptation behaviour. Nat. Clim. Chang 2019; 9:158–163.

[44] Buchs M, Sylke VS. Who emits most? associations between socio-economic factors and UK households’ home energy, transport, indirect and total CO2 emissions. Ecological Economics 2013; 90:114–123.

[45] Governoor’s Decree No. 1153 Year 2022 about Provincial Minimum Wage of Jakarta 2023.

[46] Kusumawardani D, Ajeng KD. The effect of income inequality on carbon dioxide emissions: A case study of Indonesia., Heliyon 2020; 6:1–8. doi: 10.1016/j.heliyon.2020.e04772 32904198 PMC7452505

[47] Xu X, Tan Y, Chen S, Yang G, et al. Urban household carbon emissions and contributing factors in the Yangtze River Delta, China. Plos One 2015; 10 (4): 1–21. 10.1371/journal.pone.0121604 PMC4401559 25884853

[48] Sulistyawati S, Surahma AM, Tri WS. Assessment of knowledge regarding climate change and health among adolescents in Yogyakarta, Indonesia. Journal of Environmental and Public Health 2018; 1–7. doi: 10.1155/2018/9716831 29666660 PMC5832100

[49] Yuliantoro I, Nurlita IW. Perception and adaptation of coastal community toward climate change at Sarawet Village of North Minahasa Regency. Jurnal WASIAN 2019; 6 (2): 89–99.

[50] Caparoso JK, Lagura R, Agullar MLT. Does climate change education result to less carbon? The Normal Lights 2018; 12 (1): 31–66.

[51] Tvinnereim Endre, Kjersti F, Oyvind G, Mikael PJ, Johannesson, Asta DN. 2017. Citizens’ preferences for tackling climate change. Quantitative and qualitative analyses of their freely formulated solutions. Global Environmental Change 2017; 46: 34–41.

[52] Peek L, Abramson DM, Cox RS, Fothergill A, Tobin J. Children and disasters. In H. Rodríguez W Donner J. Trainor (Eds.), Handbook of disaster research. Handbooks of sociology and social research. Berlin: Springer; 2018.

[53] Sarkawi D, Agus P, Anggi A. Environmental knowledge and environmental friendly behaviour based on gender and education level. International Journal of Advanced Research (IJAR) 2017; 5(6):2106–2113.

[54] Sanga EL. Socio-demographic determinants of access to climate change information among tomato growing farmers in Mvomero district, Tanzania’. University of Dar es Salaam Library Journal 2020; 15 (2): 121–136.

[55] Aelst KV, Holvoet N. Intersections of gender and marital status in accessing climate change adaptation: evidence from rural Tanzania. World Development 2016; 79 (C): 40–50.

[56] Jimlan JT. Carbon footprint status, climate change mitigation awareness and education for sustainable development perceptions of school principals in the Island of Panay: bases for an eco-school toolkit’. Journal of World English and Educational Practices (JWEEP) 2020; 3(1), 23–52.

[57] Kabir MI. Bayzidur R, Wayne S., et al. Knowledge and perception about climate change and human health: findings from a baseline survey among vulnerable communities in Bangladesh. BMC Public Health 2016; 16 (266): 1–10. doi: 10.1186/s12889-016-2930-3 26979241 PMC4791871

[58] Yusoff MM, Saini KM, Zainorfarah Z. Greenhouse gases awareness among local community in Simpang Empat Village, Muallim Perak. Quantum Journal of Social Sciences and Humanities 2020; 1(1): 12–22.

[59] Minister of Environment and Forestry Decree. Operational plan Indonesia’s FOLU net sink 2030. Jakarta: Minister of Environment and Forestry Decree; 2022.

[60] OECD. Decarbonizing urban mobility with land use and transport policies the case of Auckland. Paris: OECD Publishing; 2020.

[61] He X, Lin M, Chen TL, Liu B, Tseng PC, Cao W. Implementation plan for low-carbon resilient city towards Sustainable Development Goals: challenges and perspectives. Aerosol and Air Quality Research 2020; 20: 444–464.

[62] United Nations. Communication on climate change. https://www.un.org/en/climatechange/communicating-climate-change. (Accessed 15 December 2022).

[63] Ministry of National Development Planning. A green economy for a net zero future: how Indonesia can build back better after COVID-19 with the Low Carbon Development Initiative (LCDI). Jakarta: Ministry of National Development Planning; 2021.

