# Supplementary material for: The driving factors behind urban communities’ carbon emissions in the selected urban villages of Jakarta, Indonesia
Source: PLoS One. 2023 Nov 10;18(11):e0288396. doi: 10.1371/journal.pone.0288396 (PMC10637665; doi:10.1371/journal.pone.0288396)
Supplement: S1 File — (PDF) [file pone.0288396.s001.pdf]

SCRIBENDI

Certificate of Editing and Proofreading

This certifies that a version of the document titled

Driving factors of urban communities carbon emissions in the selected urban villages of Jakarta City, Indonesia

authored by

Dr. Fatmah

was edited and proofread by Scribendi as order number

939867

for clarity, consistency, and correctness according to the requirements and guidelines specified by the client.

Mon, 27 Mar 2023

Scribendi Inc

SCRIBENDI INC.
405 RIVERVIEW DRIVE
CHATHAM, ON N7M 0N3 CANADA
+1 (519) 351 1626

www.scribendi.com

Printed on 2023-03-27 at 3:17 PM
